# Misattribution bias of threat-related facial expressions is related to a longer duration of illness and poor executive function in schizophrenia and schizoaffective disorder

**DOI:** 10.1016/j.eurpsy.2007.10.004

**Published:** 2008-01

**Authors:** Preethi Premkumar, Michael A. Cooke, Dominic Fannon, Emmanuelle Peters, Tanja M. Michel, Ingrid Aasen, Robin M. Murray, Elizabeth Kuipers, Veena Kumari

**Affiliations:** a Department of Psychology, PO78, Institute of Psychiatry, King's College London, De Crespigny, Denmark Hill, London SE5 8AF, United Kingdom; b Division of Psychological Medicine, Institute of Psychiatry, King's College London, London, United Kingdom; c University Hospital Würzburg, Department of Psychiatry and Psychotherapy, Würzburg, Germany; d South London and Maudsley NHS Foundation Trust Maudsley Hospital, Denmark Hill, London, SE5 8AZ, United Kingdom

**Keywords:** Schizophrenia, Emotion, Chronic illness, Cognition

## Abstract

**Background:**

While it is known that patients with schizophrenia recognize facial emotions, specifically negative emotions, less accurately, little is known about how they misattribute these emotions to other emotions and whether such misattribution biases are associated with symptoms, course of the disorder, or certain cognitive functions.

**Method:**

Outpatients with schizophrenia or schizoaffective disorder (*n* = 73) and healthy controls (*n* = 30) performed a computerised Facial Emotion Attribution Test and Wisconsin Card Sorting Test (WCST). Patients were also rated on the Positive and Negative Syndrome Scale (PANSS).

**Results:**

Patients were poor at recognizing fearful and angry emotions and attributed fear to angry and angry to neutral expressions. Fear-as-anger misattributions were predicted independently by a longer duration of illness and WCST perseverative errors.

**Conclusion:**

The findings show a bias towards misattributing fearful and angry facial emotions. The propensity for fear-as-anger misattribution biases increases as the length of time that the disorder is experienced increases and a more rigid style of information processing is used. This, at least in part, may be perpetuated by subtle fearfulness expressed by others while interacting with people with schizophrenia.

## 1 Introduction

People with schizophrenia are characterised by an emotion recognition deficit [1,13,33,35–37]. They are less accurate at recognizing facial emotion, voice emotion [5,9,10,25] and emotion of a person from a video clip [39]. The recognition of facial emotions has been studied most extensively ([30,37], review [26]) and is influenced by illness stage with chronic patients performing worse than first-episode patients [25]. Age and time in hospital also influence it, being old and spending a longer time in hospital are associated with worse facial emotion recognition [36]. Wolwer and colleagues [42], however, did not observe worsening of facial emotion recognition over a 12-week period among patients with schizophrenia who had remitted. It is possible that the duration of illness, rather than the presence of active psychosis, plays a role. In fact, a longer duration of illness has been shown to have an adverse effect on facial emotion recognition [5,25,36] and to mediate the negative relationship between recognition of happy facial expressions and positive symptoms [36].

Earlier studies reported a negative association between emotion recognition and hallucinations and thought disorder [22], and negative symptoms [32]. More recent studies seem to suggest that facial emotion recognition deficits are independent of the experience of positive and negative symptoms [5,6,25,37] and may instead be part of a cognitive impairment, especially in the domains of executive function and attention [21,31,37].

People with schizophrenia are known to judge social situations poorly [34,39]. They recognize facial emotions, specifically negative emotions, less accurately ([24], review [26]). Little is known about how patients with schizophrenia misattribute these emotions to other facial emotions. It is possible, but yet to be established, that they display a specific pattern of misattribution. Two studies have examined this issue so far. Peer and colleagues [28] studied the association between paranoid symptoms and misattribution of happy, sad, fear and surprise emotions to the emotions of anger or disgust (misattributions to other emotions were not examined) in 91 patients with a severe mental illness (schizophrenia, schizoaffective disorder, psychosis, bipolar disorder or other DSM disorders). They found that paranoid symptoms were related to misattribution of emotions to disgust, but not to anger. In a study of 28 stable outpatients with schizophrenia and 61 healthy controls, Kohler and colleagues [24] found that patients had difficulty in recognizing fearful and neutral expressions. They also misattributed neutral expressions to negatively toned expressions, disgust and fear, more often than healthy controls.

The present study examined facial emotion recognition deficits and the clinical and cognitive correlates of misattribution of facial emotions in a large sample of outpatients with schizophrenia. Our aims were to examine in patients with schizophrenia (a) the pattern of facial emotion recognition deficits, (b) the role of duration of illness and clinical symptoms in misattribution of emotions that are recognized less accurately by patients compared to healthy individuals, and (c) the association between facial emotion misattribution and cognitive function among the schizophrenia patients. Based on previous findings, we hypothesized that patients with schizophrenia would show impairment in recognizing fear and anger. We further hypothesized that in the schizophrenia group, a higher number of emotion misattributions would be associated with (a) a longer duration of illness and (b) poor executive function.

## 2 Materials and methods

### 2.1 Participants

Participants were outpatients with a diagnosis of schizophrenia or schizoaffective disorder and healthy controls who were recruited as part of a longitudinal study on the effect of cognitive behavioural therapy (CBT) on the brain. The present investigation utilized data collected at baseline (prior to CBT).

Eighty patients were recruited to the study from the South London and Maudsley NHS Foundation Trust of whom 73 provided both emotion recognition and clinical data. Thirty of these patients were allocated to receive CBT after the baseline assessment, but none had CBT at the time of or prior to baseline assessment. Inclusion criteria were the following: a DSM-IV diagnosis of schizophrenia or schizoaffective disorder, aged between 18 and 65 years and able to speak English fluently. Patients were excluded if they had already received CBT. Clinical diagnoses were made by an experienced consultant psychiatrist (DF) using the Structured Clinical Interview for DSM-IV [14], who also administered the PANSS [20]. Thirty healthy controls were also recruited through local advertisements.

The study procedures and the use of baseline data for the purpose of the current investigation were approved by the Ethics Committee of the Joint Research Ethics Committee of the South London and Maudsley NHS Foundation Trust and the Institute of Psychiatry, London. All participants provided written informed consent to their participation in the longitudinal study on the effect of cognitive behavioural therapy (CBT) on the brain. They were compensated for their time and travel (Table 1).

### 2.2 Measures

Participants performed the computerised Facial Emotion Attribution task designed for this study which consisted of 128 pictures of male (*n* = 4) or female (*n* = 4) faces expressing neutral, happy, angry or fearful emotions [11]. Each emotion was displayed 32 times ordered pseudo-randomly to avoid repetition of a particular emotion. Each face appeared on the screen for 3 s and then disappeared. It was followed by the question: ‘What emotion did you see?’ and a list of four emotions (neutral, happy, angry and fear) from which the participant made a choice by pressing the mouse button. The faces displayed represented the “100%” emotion of that particular facial expression. The task took approximately 8 min to complete.

All patients were also examined on the Wisconsin Card Sorting Test (WCST) [16] that consistently reveals an executive function deficit in schizophrenia [17].

### 2.3 Statistical analysis

#### 2.3.1 Group differences in facial emotion attribution

The Facial Emotion Attribution task generated four emotion attribution variables (corresponding to the correct answers for each emotion type) that were used to examine the pattern of emotion recognition deficit and 12 emotion misattribution variables (corresponding to the incorrect answers for each emotion type) that were used to study a specific pattern of misattribution. The misattribution variables were neutral-as-happy, neutral-as-anger, neutral-as-fear, happy-as-neutral, happy-as-anger, happy-as-fear, anger-as-neutral, anger-as-happy, anger-as-fear, fear-as-neutral, fear-as-happy and fear-as-anger.

A two-way multivariate analysis of variance (MANOVA; Wilk's *F*) was performed with the four emotion attribution variables as the within-subjects variables and group (patients and healthy controls) as the between-subjects variable. A two-way multivariate analysis of covariance (MANCOVA) was then performed with the four emotion attribution variables as the within-subjects variables and group as the between-subjects variable to confirm any effects revealed by the MANOVA involving the group factor after covarying for age and years in education, since these two variables were found to differentiate the groups (see Section 3).

Emotion misattribution variables were analysed using MANOVA on the emotion attribution variables that revealed a deficit in the patient group, relative to the control group; the emotion attribution variables that showed no group difference had low error rates and therefore insufficient power to examine misattribution.

#### 2.3.2 Duration of illness, antipsychotic dosage, clinical and cognitive correlates of facial emotion misattribution

Spearman correlations were performed between the emotion misattribution variables (that differentiated patients from controls) and duration of illness, number of psychotic episodes, antipsychotic drug level (chlorpromazine equivalents), the PANSS total and subscale scores and WCST perseverative errors.

Where both duration of illness and WCST perseverative errors were associated with an emotion misattribution variable, a multiple regression analysis was performed to determine the amount of variance explained by each of the two predictors. In this analysis, the emotion misattribution variable was the dependent variable and WCST perseverative errors and duration of illness were the predictors entered in a stepwise fashion.

All analyses were carried out using SPSS (version 15). Alpha level for significance testing was kept at *p* = 0.05 in all analyses. No alpha corrections were used for tests of *a priori* hypotheses.

## 3 Results

### 3.1 Group differences in facial emotion attribution

**Fig. 1:**
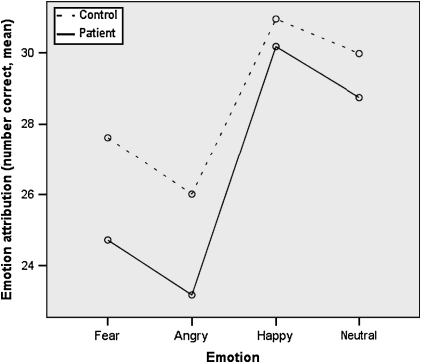
Facial emotion attributions (number correct) in patients and controls.

There was a significant main effect of group [*F*(3) = 9.30, *p* = 0.003] suggesting less accurate performance in the patient group. There was also a significant effect of emotion type [*F*(3,99) = 78.75, *p* < 0.001] showing that across both groups performance accuracy (maximum possible score = 32) was the highest for happy facial expressions, followed by neutral, fear and anger (see Fig. 1). More importantly, a significant group × emotion type interaction [*F*(3,99) = 3.19, *p* = 0.03] was observed revealing that patients were less accurate than controls at recognizing fear [*t*(101) = 2.96, *p* = 0.004] and anger [*t*(101) = 2.44, *p* = 0.004], but did not differ for happy and neutral facial expressions. The MANCOVA, controlling for age and years in education, did not eliminate the effect of group [*F*(1,99) = 6.55, *p* = 0.01], but reduced the significance of the group × emotion type interaction to a trend level [*F*(3,97) = 2.56, *p* = 0.06].

The MANOVA for group difference in misattributions (of fear and anger) was significant [*F*(6,99) = 2.23, *p* = 0.05]. Patients made significantly more fear-as-anger misattributions compared to healthy controls (see Table 2); group difference in other misattribution variables failed to survive correction for multiple comparisons.

### 3.2 Correlation between facial emotion misattribution and duration of illness

Misattributions of fear-as-anger were associated with a longer duration of illness (*r* = 0.45, *p* < 0.001).

### 3.3 Correlation between facial emotion misattribution and number of psychotic episodes, antipsychotic dosage and clinical symptoms

Misattributions of fear-as-anger and anger-as-neutral were not significantly associated with the number of psychotic episodes, antipsychotic drug level, PANSS total score or positive, negative and general psychopathology subscale scores (max *r* value = 0.22).

### 3.4 Correlation between facial emotion misattribution and cognitive function in patients

The number of misattributions of fear-as-anger correlated with WCST perseverative errors (*r* = 0.32, *p* = 0.005).

### 3.5 Multiple regression findings

We tested the independent contribution of duration of illness and WCST perseverative errors towards explaining the variance in fear-as-anger misattribution. Both duration of illness and WCST perseverative errors predicted fear-as-anger misattribution [*F*(2,71) = 12.99, *p* < 0.001]. Duration of illness and WCST perseverative errors predicted 16% and 10% of the misattribution variance, respectively.

## 4 Discussion

The key findings from this study were the following: (a) across groups, happy facial expressions were recognized most accurately, followed by neutral, then fearful and angry expressions; (b) patients were less accurate than controls in recognizing fearful and angry facial expressions; (c) patients, relative to controls, made more fear-as-anger misattributions; and (d) a longer duration of illness and perseverative errors on the WCST were independently associated with misattributions of fearful expressions as anger.

The present observation of poorer recognition of negative emotions than of positive emotions across both groups is consistent with previous literature [25]. The patients, as expected, showed further difficulty in recognizing negative emotions relative to the healthy controls. Our finding of poorer recognition of fearful and angry facial emotions among schizophrenia patients is also consistent with evidence from a predominant facial emotion recognition deficit of threat-related expressions [10,26].

Our finding of a relationship between duration of illness and number of emotion misattributions of fear-as-anger is in line with our hypothesis. It supports previous reports of an association between a longer duration of illness and poorer accuracy of facial emotion recognition [5,25,36], but more importantly, reveals that this association is particularly relevant to fear-as-anger misattributions. Most of our patients were of the paranoid type, and it is already known that paranoid individuals tend to judge facial emotions as angry [38]. Our findings suggest that the propensity for such biases increases the longer the psychiatric disorder is experienced.

The absence of an association between PANSS symptoms and misattributions of facial emotional expressions corroborates earlier findings of no association between accuracy of facial emotion recognition and positive or negative symptoms [4,5,2,31,37]. Peer and colleagues [28] suggest that an emotion misattribution bias is exacerbated by a poor ability to shift set, i.e., a perseverative response style. Our study showed that the number of WCST perseverative errors was positively associated with fear-as-anger misattributions. This finding suggests that such misattributions, at least to some extent, are cognitively mediated and that schizophrenia individuals may be adopting a rigid response style possibly perpetuated by fear among others while interacting with people with schizophrenia. Our analyses did not show an effect of age, pre-morbid IQ, number of psychotic episodes and medication on fear-as-anger or anger-as-neutral misattributions. It is, however, possible that other factors, such as duration of untreated psychosis, had some influence on our findings.

Increased affect misattributions among people with psychosis may also relate to an abnormal causal attribution style. Paranoid patients are known to make excessively external attributions for negative events relative to depressed and normal controls [19] and relative to Asperger's syndrome and normal controls [7]. Patients with persecutory and grandiose beliefs are reported to show an externalising attributional style for negative events [18]. Such a self-preserving attributional style may function to protect self-esteem [3].

At a neural level, increased misattributions of fear-as-anger may reflect an altered fronto-mesiotemporal circuit [40]. A number of imaging studies on emotional recognition in schizophrenia indicate abnormality relating to processing of negative emotions [23,30]. Williams and colleagues [40] observed greater impairment in recognition of fear in patients with paranoid schizophrenia. They reported that paranoid schizophrenia is characterised by a specific disjunction of arousal and amygdala–prefrontal circuits in relation to recognition of fearful facial expressions. This disjunction appears to be most apparent in patients with a profile of paranoia, coupled with poor social function and insight [41] and is present in both the conscious and unconscious processings of fearful facial expressions [8]. Supporting amygdala dysfunction further in paranoid schizophrenia, a recent functional MRI study involving viewing of dynamic fearful faces observed increased bilateral amygdala activation relative to healthy controls in paranoid, but not in non-paranoid, patients with schizophrenia [30]. The reduction in prefrontal cortical volume with a longer duration of illness [29] may lead to a loss of the prefrontal cortical function to regulate amygdala-autonomic function. It would be interesting to know whether such facial emotional misattributions are associated with differential activation of the prefrontal cortex and amygdala in patients with schizophrenia.

Our study has a number of limitations. First, it is difficult to separate the effect of duration of illness from that of ageing [15], because the longitudinal effects of psychiatric illness unfold with age. Age, however, did not have a significant influence on emotion attribution/misattribution in our task in the group of healthy people (data not shown). Second, our experimental paradigm did not include sad and disgust emotions that would have informed us about the extent of misattribution of negative facial emotions. All faces were of Caucasians that may have disadvantaged participants from other ethnic groups. People are better at recognizing facial emotions among people from their own racial group [12], though this may not have been a factor in the particular emotion misattribution patterns seen in our study. Finally, this study used duration of illness as a measure of longitudinal illness process in a cross-sectional design which may be prone to a selection bias, since patients with a better outcome may not remain in contact with mental health services. The association we find between the longer duration of illness and emotion misattribution may therefore also include the contribution of other variables such as a higher genetic predisposition possibly leading to a type of illness with a chronic course. This possibility needs to be examined in future prospective studies. Cross-sectional studies, however, generally serve as a prelude to longitudinal prospective studies.

In conclusion, the present findings confirm that patients with schizophrenia have a bias towards misattributing negative facial emotions, especially fear-as-anger, and suggest that the propensity for such biases increases the longer the disorder is experienced. The association between misattributions of fear-as-anger expressions and WCST perseverative errors may suggest that a rigid response style in response to fearfulness is likely to be expressed by those around them.

## Figures and Tables

**Table 1:** Participant characteristics

	Patient (*n* = 73)	Control (*n* = 30)	*t* or *χ*^2^(df)	*p*-Value
Age, years – mean (SD)	38.58 (9.93)	33.17 (11.82)	2.37 (101)	0.02
Years in education – mean (SD)	13.66 (2.50)	15.30 (2.55)	2.99 (101)	0.003
Pre-morbid IQ^a^ – mean (SD)	107.33 (9.55)	114.37 (8.54)	8.54 (101)	0.001
Sex – male/female (*n*)	52/21	20/10	0.21	0.37
Ethnicity				
White	27	20		
Afro-Caribbean	30	5		
Asian	5	1		
Mixed race	5	3		
Other	6	1		

*Patient characteristics*				
Diagnosis (*n*)				
Schizophrenia				
Paranoid	58			
Residual	3			
Catatonic	1			
Disorganised	1			
Unclassified	3			
Schizoaffective disorder	7			
Age of onset, years – mean (SD)	24.05 (7.66)			
Duration of illness, years – mean (SD)	14.60 (10.02)			
Number of psychotic episodes – mean (SD) (*n* = 40)	3.35 (3.19)			

*PANSS – mean (SD)*				
Positive	16.93 (4.75)			
Negative	18.18 (4.63)			
General psychopathology	32.88 (6.60)			
Total	67.99 (13.26)			

*Current antipsychotic medication type*				
Atypical	60			
Typical	11			
Non-compliant	2			
Antipsychotic medication dosage (chlorpromazine equivalents)	489.70 (336.73)			

a Pre-morbid IQ estimated by the National Adult Reading Test [27].

**Table 2:** Number of facial emotion misattributions in patients and healthy controls

	Patients	Controls	*F*-value	*p*-Value
Mean (SD)	Mean (SD)
Fear-as-anger^a^	3.55 (5.00)	0.97 (1.54)	**7.95**	**0.006**^b^
Fear-as-happy^a^	0.71 (1.31)	0.27 (0.83)	3.45	0.06
Fear-as-neutral	1.04 (1.84)	0.7 (1.62)	1.50	0.22
Anger-as-fear	3.04 (2.98)	2.30 (2.38)	1.29	0.26
Anger-as-happy^a^	0.70 (1.58)	0.23 (0.50)	2.86	0.09
Anger-as-neutral^a^	2.81 (2.95)	1.73 (2.07)	4.51	0.04

a Equal variances not assumed.

b Significant after correction (Bonferroni) for multiple comparisons.

## References

[1] Abdi Z., Sharma T. (2004). Social cognition and its neural correlates in schizophrenia and autism. CNS Spectr.

[2] Addington J., Addington D. (1998). Facial affect recognition and information processing in schizophrenia and bipolar disorder. Schizophr Res.

[3] Bentall R.P., Corcoran R., Howard R., Blackwood N., Kinderman P. (2001). Persecutory delusions: a review and theoretical integration. Clin Psychol Rev.

[4] Borod J.C., Martin C.C., Alpert M., Brozgold A., Welkowitz J. (1993). Perception of facial emotion in schizophrenic and right brain-damaged patients. J Nerv Ment Dis.

[5] Bozikas V.P., Kosmidis M.H., Anezoulaki D., Giannakou M., Karavatos A. (2004). Relationship of affect recognition with psychopathology and cognitive performance in schizophrenia. J Int Neuropsychol Soc.

[6] Brune M. (2005). Emotion recognition, ‘theory of mind,’ and social behavior in schizophrenia. Psychiatry Res.

[7] Craig J.S., Hatton C., Craig F.B., Bentall R.P. (2004). Persecutory beliefs, attributions and theory of mind: comparison of patients with paranoid delusions, Asperger's syndrome and healthy controls. Schizophr Res.

[8] Das P., Kemp A.H., Flynn G., Harris A.W., Liddell B.J., Whitford T.J. (2007). Functional disconnections in the direct and indirect amygdala pathways for fear processing in schizophrenia. Schizophr Res.

[9] de Gelder B., Vroomen J., de Jong S.J., Masthoff E.D., Trompenaars F.J., Hodiamont P. (2005). Multisensory integration of emotional faces and voices in schizophrenics. Schizophr Res.

[10] Edwards J., Pattison P.E., Jackson H.J., Wales R.J. (2001). Facial affect and affective prosody recognition in first-episode schizophrenia. Schizophr Res.

[11] Ekman P., Friesen W.V. (1976). Pictures of facial affect.

[12] Elfenbein H.A., Ambady N. (2002). On the universality and cultural specificity of emotion recognition: a meta-analysis. Psychol Bull.

[13] Feinberg T.E., Rifkin A., Schaffer C., Walker E. (1986). Facial discrimination and emotional recognition in schizophrenia and affective disorders. Arch Gen Psychiatry.

[14] First M.B., Spitzer R.L., Gibbon M., Williams J.B.W. (2002). Structured clinical interview for DSM-IV-TR Axis I disorders.

[15] Friedman J.I., Harvey P.D., Coleman T., Moriarty P.J., Bowie C., Parrella M. (2001). Six-year follow-up study of cognitive and functional status across the lifespan in schizophrenia: a comparison with Alzheimer's disease and normal aging. Am J Psychiatry.

[16] Heaton R.K. (1981). Wisconsin Card Sorting Test.

[17] Heinrichs R.W., Zakzanis K.K. (1998). Neurocognitive deficits in schizophrenia: a quantitative review of the evidence. Neuropsychology.

[18] Jolley S., Garety P., Bebbington P., Dunn G., Freeman D., Kuipers E. (2006). Attributional style in psychosis – the role of affect and belief type. Behav Res Ther.

[19] Kaney S., Bentall R.P. (1989). Persecutory delusions and attributional study. Br J Med Psychol.

[20] Kay S.R., Fiszbein A., Opier L.A. (1987). The Positive and Negative Syndrome Scale (PANSS) for schizophrenia. Schizophr Bull.

[21] Kerr S.L., Neale J.M. (1993). Emotion perception in schizophrenia: specific deficit or further evidence of generalized poor performance?. J Abnorm Psychol.

[22] Kohler C.G., Bilker W., Hagendoorn M., Gur R.E., Gur R.C. (2000). Emotion recognition deficit in schizophrenia: association with symptomatology and cognition. Biol Psychiatry.

[23] Kohler C.G., Martin E.A. (2006). Emotional processing in schizophrenia. Cogn Neuropsychiatry.

[24] Kohler C.G., Turner T.H., Bilker W.B., Brensinger C.M., Siegel S.J., Kanes S.J. (2003). Facial emotion recognition in schizophrenia: intensity effects and error pattern. Am J Psychiatry.

[25] Kucharska-Pietura K., David A.S., Masiak M., Phillips M.L. (2005). Perception of facial and vocal affect by people with schizophrenia in early and late stages of illness. Br J Psychiatry.

[26] Mandal M.K., Pandey R., Prasad A.B. (1998). Facial expressions of emotions and schizophrenia: a review. Schizophr Bull.

[27] Nelson H.E., Wilson J. (1991). National adult reading test manual.

[28] Peer J.E., Rothmann T.L., Penrod R.D., Penn D.L., Spaulding W.D. (2004). Social cognitive bias and neurocognitive deficit in paranoid symptoms: evidence for an interaction effect and changes during treatment. Schizophr Res.

[29] Premkumar P., Kumari V., Corr P.J., Sharma T. (2006). Frontal lobe volumes in schizophrenia: effects of stage and duration of illness. J Psychiatr Res.

[30] Russell T.A., Reynaud E., Kucharska-Pietura K., Ecker C., Benson P.J., Zelaya F. (2007). Neural responses to dynamic expressions of fear in schizophrenia. Neuropsychologia.

[31] Salem J.E., Kring A.M., Kerr S.L. (1996). More evidence for generalized poor performance in facial emotion perception in schizophrenia. J Abnorm Psychol.

[32] Schneider F., Gur R.C., Gur R.E., Shtasel D.L. (1995). Emotional processing in schizophrenia: neurobehavioral probes in relation to psychopathology. Schizophr Res.

[33] Schneider F., Gur R.C., Koch K., Backes V., Amunts K., Shah N.J. (2006). Impairment in the specificity of emotion processing in schizophrenia. Am J Psychiatry.

[34] Sergi M.J., Green M.F. (2003). Social perception and early visual processing in schizophrenia. Schizophr Res.

[35] Silver H., Goodman C., Knoll G., Isakov V., Modai I. (2005). Schizophrenia patients with a history of severe violence differ from nonviolent schizophrenia patients in perception of emotions but not cognitive function. J Clin Psychiatry.

[36] Silver H., Shlomo N., Turner T., Gur R.C. (2002). Perception of happy and sad facial expressions in chronic schizophrenia: evidence for two evaluative systems. Schizophr Res.

[37] Silver H., Shlomo N. (2001). Perception of facial emotions in chronic schizophrenia does not correlate with negative symptoms but correlates with cognitive and motor dysfunction. Schizophr Res.

[38] Smari J., Stefansson S., Thorgilsson H. (1994). Paranoia, self-consciousness, and social cognition in schizophrenics. Cogn Ther Res.

[39] Toomey R., Schuldberg D., Corrigan P., Green M.F. (2002). Nonverbal social perception and symptomatology in schizophrenia. Schizophr Res.

[40] Williams L.M., Das P., Harris A.W.F., Liddell B.B., Brammer M.J., Olivieri G. (2004). Dysregulation of arousal and amygdala–prefrontal systems in paranoid schizophrenia. Am J Psychiatry.

[41] Williams L.M., Das P., Liddell B.J., Olivieri G., Peduto A.S., David A.S. (2007). Fronto-limbic and autonomic disjunctions to negative emotion distinguish schizophrenia subtypes. Psychiatry Res.

[42] Wolwer W., Streit M., Polzer U., Gaebel W. (1996). Facial affect recognition in the course of schizophrenia. Eur Arch Psychiatry Clin Neurosci.

